# Entry Tropism of BK and Merkel Cell Polyomaviruses in Cell Culture

**DOI:** 10.1371/journal.pone.0042181

**Published:** 2012-07-31

**Authors:** Rachel M. Schowalter, William C. Reinhold, Christopher B. Buck

**Affiliations:** 1 Tumor Virus Molecular Biology Section, Laboratory of Cellular Oncology, National Cancer Institute, Bethesda, Maryland, United States of America; 2 Laboratory of Molecular Pharmacology, Center for Cancer Research, National Cancer Institute, Bethesda, Maryland, United States of America; University Hospital Hamburg-Eppendorf, Germany

## Abstract

Merkel Cell Polyomavirus (MCV or MCPyV) was recently discovered in an aggressive form of skin cancer known as Merkel cell carcinoma (MCC). Integration of MCV DNA into the host genome likely contributes to the development of MCC in humans. MCV infection is common and many healthy people shed MCV virions from the surface of their skin. MCV DNA has also been detected in samples from a variety of other tissues. Although MCC tumors serve as a record that MCV can infect the Merkel cell lineage, the true tissue tropism and natural reservoirs of MCV infection in the host are not known. In an effort to gain insight into the tissue tropism of MCV, and to possibly identify cellular factors responsible for mediating infectious entry of the virus, the infection potential of human cells derived from a variety of tissues was evaluated. MCV gene transfer vectors (pseudoviruses) carrying reporter plasmid DNA encoding GFP or luciferase genes were used to transduce keratinocytes and melanocytes, as well as lines derived from MCC tumors and the NCI-60 panel of human tumor cell lines. MCV transduction was compared to transduction with pseudoviruses based on the better-studied human BK polyomavirus (BKV). The efficiency of MCV and BKV transduction of various cell types occasionally overlapped, but often differed greatly, and no clear tissue type preference emerged. Application of native MCV virions to a subset of highly transducible cell types suggested that the lines do not support robust replication of MCV, consistent with recent proposals that the MCV late phase may be governed by cellular differentiation in vivo. The availability of carefully curated gene expression data for the NCI-60 panel should make the MCV and BKV transduction data for these lines a useful reference for future studies aimed at elucidation of the infectious entry pathways of these viruses.

## Introduction

Polyomaviruses have a long history as suspected agents underlying various cancers in humans. However, not until the discovery of Merkel cell polyomavirus (MCV or MCPyV) in a rare form of skin cancer, known as Merkel cell carcinoma (MCC), has conclusive evidence been brought in support of a causal relationship of a polyomavirus to cancer in human populations. Diagnosis of MCC is infrequent, with about 1,500 cases identified each year in the United States [1]. Nevertheless, like other human polyomaviruses, such as BK polyomavirus (BKV or BKPyV), infection by MCV appears to be widespread. A large majority of the adult population has developed antibodies against both viruses [2], [3], [4], [5], [6]. BKV was discovered more than four decades ago in the urine of a kidney transplant recipient [7]. It soon became clear that nearly all humans harbor asymptomatic BKV infections in their urinary epithelium [8], [9], [10]. Although BKV can cause cancer in experimentally-exposed animals, conclusive evidence of a fundamental role for BKV as a causal agent underlying human cancer is lacking (reviewed in [11]). On the other hand, BKV is frequently a serious threat to certain organ transplant recipients undergoing immune suppressive therapy. Most notably, BKV-induced nephropathy drastically increases the risk of graft failure in 1–10% of kidney transplant recipients [12].

The primary site of MCV replication in humans is not known. Although MCV DNA is found clonally integrated in MCCs [13], the MCV genomic DNA in tumors typically carries mutations that would prevent virus replication [14]. It is not known whether primary Merkel cells or their precursors can be productively infected by MCV or are instead merely a “bystander” cell type. in vitro culture of primary human Merkel cells has not yet been reported. Merkel cells are found in the basal layer of the skin and mucosa where they typically associate with sensory axons (reviewed in [15]). Although MCV has been detected in abundance from healthy human skin swabs [16], [17], [18], it is uncertain which of the dozen or so different cell types that make up the skin are the source of MCV virions. Furthermore, MCV DNA has also been detected in respiratory samples [19], [20], [21], urine [22], and blood [23], [24]. Thus, the precise cellular tropism of MCV is not understood.

Non-enveloped DNA viruses, such as BKV and MCV, must engage a variety of cellular factors during the infectious entry process. Direct association with an appropriate cellular receptor (or receptors) that mediates attachment and entry is an essential first step in this process. Attachment of MCV to cell surfaces was recently shown to require glycosaminoglycans, such as heparan sulfate [25]. The presence of a co-receptor glycan containing sialic acid is also hypothesized to exist, since MCV could bind but not infect cells with a defect in sialylated glycan production [25]. The sialylated glycolipids GT1b and GD1b are known to mediate BKV attachment and entry into tissue cultured cells, and cells that lack these complex gangliosides are resistant to BKV infection [26]. The urinary epithelium that BKV infects in vivo has also been shown to express these molecules [27]. While expression of the appropriate attachment receptors and co-receptors is likely an essential determinant of polyomavirus tissue tropism in vivo, post-attachment infectious entry events are also dependent on cellular factors and may therefore restrict tissue tropism as well. Compared to other polyomaviruses, such as SV40, MCV replicates very poorly in conventional monolayer cell cultures [25], [28], [29]. Lab-adapted BKV strains containing a rearranged non-coding control region (NCCR) can be efficiently propagated in cell culture. However, only recently has a model for propagation of primary BKV isolates been developed [30]. This advancement was enabled by the stable expression of SV40 T antigens, which drive BKV DNA replication and late protein expression. We have previously developed a similar method for propagation of native MCV in culture through stable expression of MCV early proteins (small t antigen and large T antigen) in 293TT cells [25]. We have also developed BKV and MCV based gene transfer vectors (pseudoviruses) capable of delivering reporter genes to cultured cells. These pseudoviruses effectively bypass the post-entry blocks on BKV and MCV replication and allow quantitation of the transducibility of cell lines that do not support the full viral life cycle.

A bioinformatics approach that utilizes the NCI-60 panel of human tumor cell lines has been used successfully by multiple groups to discover viral receptors and other cellular factors required for efficient viral infection [31], [32], [33], [34], [35]. The NCI-60 comprises sixty different cell lines originating from cancers of the lung, colon, brain, ovary, breast, prostate, kidney, as well as leukemia and melanoma lines. The panel is maintained by the Developmental Therapeutics Program of the National Cancer Institute for use in anticancer drug discovery (http://dtp.cancer.gov). The power of the NCI-60 lies in the extensive characterization of the cells in the panel, including comprehensive gene expression profiling data [36]. This allows for correlation of viral infection levels with transcript levels of a comprehensive set of genes. Previous analyses suggest that gene transcript levels in these cells significantly correlate with protein levels 65% of the time. [37]. While it is tempting to speculate that infectivity of a tumor cell line from a particular organ is indicative of potential infection of that organ in vivo, an important caveat to this approach is highlighted by a recent study suggesting that various tumor cell lines grown in culture are in many ways more similar to other cultured cells than they are to cells resident in the tissue of origin [38].

In an effort to gain insight into the tissue tropism of MCV, and to possibly identify cellular factors that are responsible for mediating entry of the virus, titers for MCV and BKV pseudoviruses were determined on the entire NCI-60 panel of cell lines. The resulting titers on these cells spanned several orders of magnitude, and revealed no clear preference for tissue of origin. Titers determined with pseudovirus-mediated delivery of plasmid DNA encoding GFP were verified with a second challenge using a pseudovirus carrying a *Gaussia* luciferase reporter gene. MCV and BKV efficiently transduced many of the same cell types, but also many distinct cell types. Bioinformatics analysis of the infectivity data with the NCI-60 gene expression data revealed many strong gene expression correlations with transducibility of the various lines. Other cell lines and primary cells of particular interest were analyzed for their capacity to support infectious entry as well. The ability of multiple highly transducible cell types to support replication of MCV genomes delivered via native MCV virions was also examined, and the results confirm that MCV replication is highly restricted in cultured cells.

**Table 1:** Relative transducibility of NCI-60 Cell Lines.

			Viral Titer		RLUs (% of A549)	
Cell Line Name	Inoculation Density	Cancer of Origin	MCV-GFP	BKV-GFP	MCV-GLuc	BKV-GLuc
CCRF-CEM	40000	Leukemia	18540	12593	0.31	ND
HL-60(TB)	40000	Leukemia	53698	52338	0.00	ND
K-562	5000	Leukemia	32175	82938	1.54	ND
MOLT-4	30000	Leukemia	0	0	0.06	ND
RPMI-8226	20000	Leukemia	61645	132552	4.56	ND
SR	20000	Leukemia	72965	653293	1.32	ND
A549/ATCC	7500	Non-Small Cell Lung Cancer	4036276	4694740	100.00	100.00
EKVX	20000	Non-Small Cell Lung Cancer	551394	1850527	ND	ND
EKVX	10000	Non-Small Cell Lung Cancer	343382	4425743	52.84	285.05
HOP-62	10000	Non-Small Cell Lung Cancer	70724	57185	2.80	1.01
HOP-92	20000	Non-Small Cell Lung Cancer	1545942	367962	12.81	ND
NCI-H226	20000	Non-Small Cell Lung Cancer	54044	21543344	2.46	ND
NCI-H23	20000	Non-Small Cell Lung Cancer	481240	564377	42.41	ND
NCI-H322M	20000	Non-Small Cell Lung Cancer	297805	151958	10.17	ND
NCI-H460	7500	Non-Small Cell Lung Cancer	30022	224570	3.51	ND
NCI-H522	20000	Non-Small Cell Lung Cancer	121885	1025356	3.27	27.13
COLO 205	15000	Colon Cancer	7591	9938	0.25	ND
HCC-2998	15000	Colon Cancer	33965	197907	0.27	2.10
HCT-116	5000	Colon Cancer	92748	4969471	6.51	190.19
HCT-15	10000	Colon Cancer	64842	0	0.72	0.85
HT29	5000	Colon Cancer	28156	1363256	1.43	ND
KM12	15000	Colon Cancer	37477	84911	1.15	6.58
SW-620	10000	Colon Cancer	36340	4102580	0.72	ND
SF-268	15000	CNS Cancer	304884	1492174	32.82	53.03
SF-295	10000	CNS Cancer	46983	2970000	1.93	ND
SF-539	15000	CNS Cancer	3563441	4433878	18.20	33.04
SNB-19	15000	CNS Cancer	391984	1270160	3.80	36.50
SNB-75	20000	CNS Cancer	438260	4097809	2.83	ND
U251	7500	CNS Cancer	48585	977771	0.36	ND
LOX IMVI	7500	Melanoma	54527	491927	0.56	ND
MALME-3M	20000	Melanoma	6350615	123229	285.04	1.41
M14	15000	Melanoma	252053	276480	31.60	ND
SK-MEL-2	20000	Melanoma	4325015	1454820	659.79	ND
SK-MEL-28	10000	Melanoma	2064602	0	34.50	6.27
SK-MEL-5	10000	Melanoma	4690038	37667	558.26	ND
UACC-257	20000	Melanoma	889504	160761	383.18	ND
UACC-62	10000	Melanoma	3174044	523162	328.73	47.71
MDA-MB-435*	15000	Melanoma	3807258	913337	228.80	ND
IGR-OV1	10000	Ovarian Cancer	188042	1156548	0.77	7.87
OVCAR-3	10000	Ovarian Cancer	3806616	4700732	133.37	ND
OVCAR-4	10000	Ovarian Cancer	56135	18150671	52.23	527.06
OVCAR-5	20000	Ovarian Cancer	49767	16918	0.03	1.20
OVCAR-8	10000	Ovarian Cancer	2199023	16645456	33.76	361.46
SK-OV-3	20000	Ovarian Cancer	35549	1526071	0.45	6.66
NCI/ADR-RES**	15000	Ovarian Cancer	17084329	25828518	571.62	448.29
786–0	10000	Renal Cancer	63979	76850	0.35	ND
A498	25000	Renal Cancer	203031	2436377	ND	ND
ACHN	10000	Renal Cancer	20727	112273	0.47	0.61
CAKI-1	10000	Renal Cancer	1826882	960036	41.42	7.54
RXF 393	15000	Renal Cancer	63262	10027078	0.09	ND
SN12C	15000	Renal Cancer	608850	1487265	16.00	37.41
TK-10	15000	Renal Cancer	464909	2059778	1.13	14.02
UO-31	15000	Renal Cancer	156469	130502	2.05	1.37
PC-3	7500	Prostate Cancer	175126	106887	10.74	ND
DU-145	10000	Prostate Cancer	2042776	270406	46.76	ND
MCF7	10000	Breast Cancer	564819	2684677	40.18	69.90
MDA-MB-231/ATCC	20000	Breast Cancer	923625	942535	28.71	18.55
HS 578T	20000	Breast Cancer	126749	449933	ND	ND
HS 578T	5000	Breast Cancer	1217837	5645014	19.86	ND
MDA-MB-468	20000	Breast Cancer	4386206	706258	236.97	19.21
BT-549	20000	Breast Cancer	29341	131896	0.30	ND
T-47D	20000	Breast Cancer	269198	46503560	4.43	ND

The cells listed in column one were plated in 96-well plates at the density shown the day prior to addition of MCV or BKV pseudoviruses. The viral titer of GFP-reporter pseudoviruses was determined by flow cytometry. GLuc-reporter transduction was measured by luminometry following injection of substrate. Relative light units (RLUs) are displayed as a percentage of A549 cell transduction RLUs. ND =  not determined.

* Once considered a breast cancer cell line, studies have shown that MDA-MB-435 cells were derived from the M14 melanoma cell line, and the gene expression profile of these cells resembles that of other melanoma cells. **Once considered a breast cancer cell line, studies have shown that NCI/ADR-RES cells were derived from the OVCAR-8 ovarian cancer cell line, and the gene expression profile of these cells resembles that of OVCAR-8.

## Methods

### Reporter Vector Production and Purification

MCV and BKV reporter vector (pseudovirus) stocks were produced using methods reported previously [4], [25]. For MCV capsid production, 293 TT cells [39] were transfected with the plasmids pwM2m [40] and ph2m [4], which express codon-modified versions of the VP1 and VP2 genes of MCV strain 339. BKV production used a mixture of four plasmids, pwB2b pwB3b, ph2b and ph3b [25], which carry codon-modified versions of the capsid proteins of BKV genotype IV isolate A-66H. For GFP reporter viruses, the capsid protein plasmids were co-transfected with an equal mixture of the plasmids pYafw [39] and pEGFP-N1 (Clontech), which utilize recombinant EF1α or CMV immediate early promoters, respectively. *Gaussia* luciferase reporter viruses instead used a mixture of the plasmids phGluc [4](EF1α promoter) and pCGluc (CMV promoter), which contain the gene encoding *Gaussia* luciferase (NEB). Forty-eight hours after transfection, the cells were harvested and lysed in Dulbecco’s phosphate buffered saline (DPBS, Invitrogen) supplemented with 9.5 mM MgCl_2_, 25 mM ammonium sulfate (starting from a 1 M stock solution adjusted to pH 9), antibiotic-antimycotic (Invitrogen), 0.5% Triton X-100 (Pierce) and 0.1% RNase A/T1 cocktail (Ambion). The cell lysate was incubated at 37°C overnight to promote capsid maturation [41]. Lysates containing mature capsids were then clarified by centrifugation for 10 min at 5000×g twice. The clarified supernatant was loaded onto a 27–33–39% iodixanol (Optiprep, Sigma) step gradient prepared in DPBS with a total of 0.8 M NaCl. The gradients were ultracentrifuged 3.5 hours in an SW55 rotor at 50,000 rpm (234,000×g). Gradient fractions were screened for the presence of encapsidated DNA using Quant-iT Picogreen dsDNA Reagent (Invitrogen). Detailed methods and maps of plasmids used in this work can be found on our lab website <http://home.ccr.cancer.gov/Lco/>.

### Cells

The NCI-60 panel of human tumor lines was purchased from the Developmental Therapeutics Program (DTP; National Cancer Institute, NIH). Each of these lines was cultured as directed by the DTP using the recommended medium, RPMI 1640 (Invitrogen) supplemented with 5% FBS (HyClone) and 1 mM L-glutamine (Hyclone). The MCC cell lines (WaGa [42], MaTi [42], UISO [43], and MKL-1 [44]) were kindly provided by Jürgen C. Becker (Medical University of Graz, Austria). The MCC cells and PFSK-1 cells (ATCC) were maintained in RPMI 1640 (Invitrogen) supplemented with 10% FBS (Sigma), Glutamax-I (Invitrogen) and MEM non-essential amino acids (Invitrogen). HEKa (human epidermal keratinocytes, adult) and HEMn (human epidermal melanocytes, neonatal) were purchased from Invitrogen and maintained in Medium 254 supplemented with either HKGS (HEKa) or HMGS-2 (HEMn). HeLa (ATCC) and HaCaT cells were maintained in DMEM (Invitrogen) with 10% FBS (Sigma), Glutamax-I and MEM non-essential amino acids (D10 medium). HaCaT cells were the generous gift of Nobert Fusenig [45]. 293TT cells were maintained in D10 medium supplemented with hygromycin (250 µg/ml; Roche) and 293–4 T cells [25] were maintained in D10 medium supplemented with zeocin (100 µg/ml; Invitrogen) and blasticidin S (5 µg/ml; Invitrogen).

**Figure 1:**
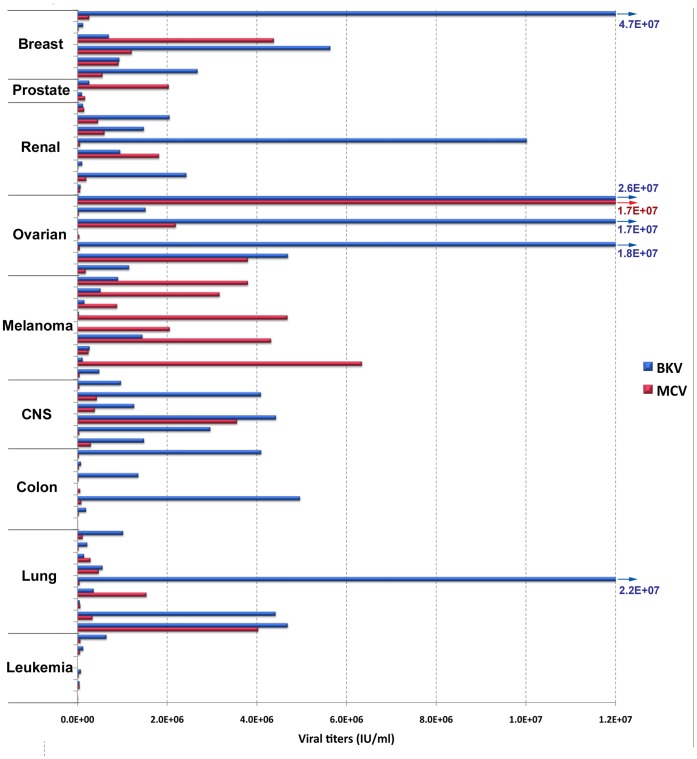
Transducivity of NCI-60 Cell Lines. A graphical representation of the GFP-reporter pseudovirus titers listed in Table 1.

### Cell Transduction Experiments

The NCI-60 panel of cell lines were plated in a 96 well plate at the density specified on the DTP website (plating density is listed in Table 1). In most instances, this resulted in a subconfluent monolayer approximately 20 hours later, when the reporter pseudovirus was added. In instances when this plating density resulted in a visibly confluent layer of cells, the plating and infection was repeated at a lower density. In cases where the plating density appeared to make a difference in MCV titer, both values were reported in Table 1. Non-NCI-60 cell types were also plated the day prior to addition of pseudovirus. The number of cells needed to result in a 30–50% confluent monolayer was determined empirically and stated and Table 1. Transduction experiments were normally performed in groups of 6 to 12 cell lines at once. In order to set a standard for experimental daily variation and provide a positive control, A549 cells, which were initially found to be relatively transducible with both MCV and BKV, were always plated and transduced side-by-side with other cells in an experiment. Five doses of a two-fold dilution series of each virus stock was inoculated onto each cell type. The third dilution (middle dose) of each virus resulted in detectable GFP transduction of ∼5–15% of A549 cells at the time of analysis. Cells were plated in 50 µl/well of growth medium and virus was added in an additional 50 µl/well medium. To minimize plate edge effects, the outer wells of the plate were not used for the assay and were instead filled with culture medium. To measure viral transduction of the GFP gene, approximately 72 hrs post-inoculation, adherent cells were incubated with trypsin to detach them from the plate and transferred to an untreated 96 well plate and suspended in wash medium (WM; DPBS with 1% FBS, antibiotic-antimycotic, and 10 mM HEPES, pH 8). Cells grown in suspension were simply transferred to the untreated plate and diluted in WM. Cells were then analyzed by flow cytometery for GFP reporter gene expression in a FACS Canto II with HTS (BD Biosciences). For measurement of *Gaussia* luciferase expression, approximately 72 hrs post-inoculation, the plate containing cells was agitated and 25 µl of conditioned culture supernatant was transferred to a white 96-well luminometry plate (Perkin Elmer). A BMG Labtech Polarstar Optima luminometer was used to inject 50 µl of *Gaussia* Luciferase Assay Kit substrate (NEB), and light emission (in relative light units, RLUs) was measured according to manufacturer instructions. The middle dose of virus on A549 cells typically resulted in 150,000–200,000 RLUs with a background of ∼500 RLUs.

**Figure 2:**
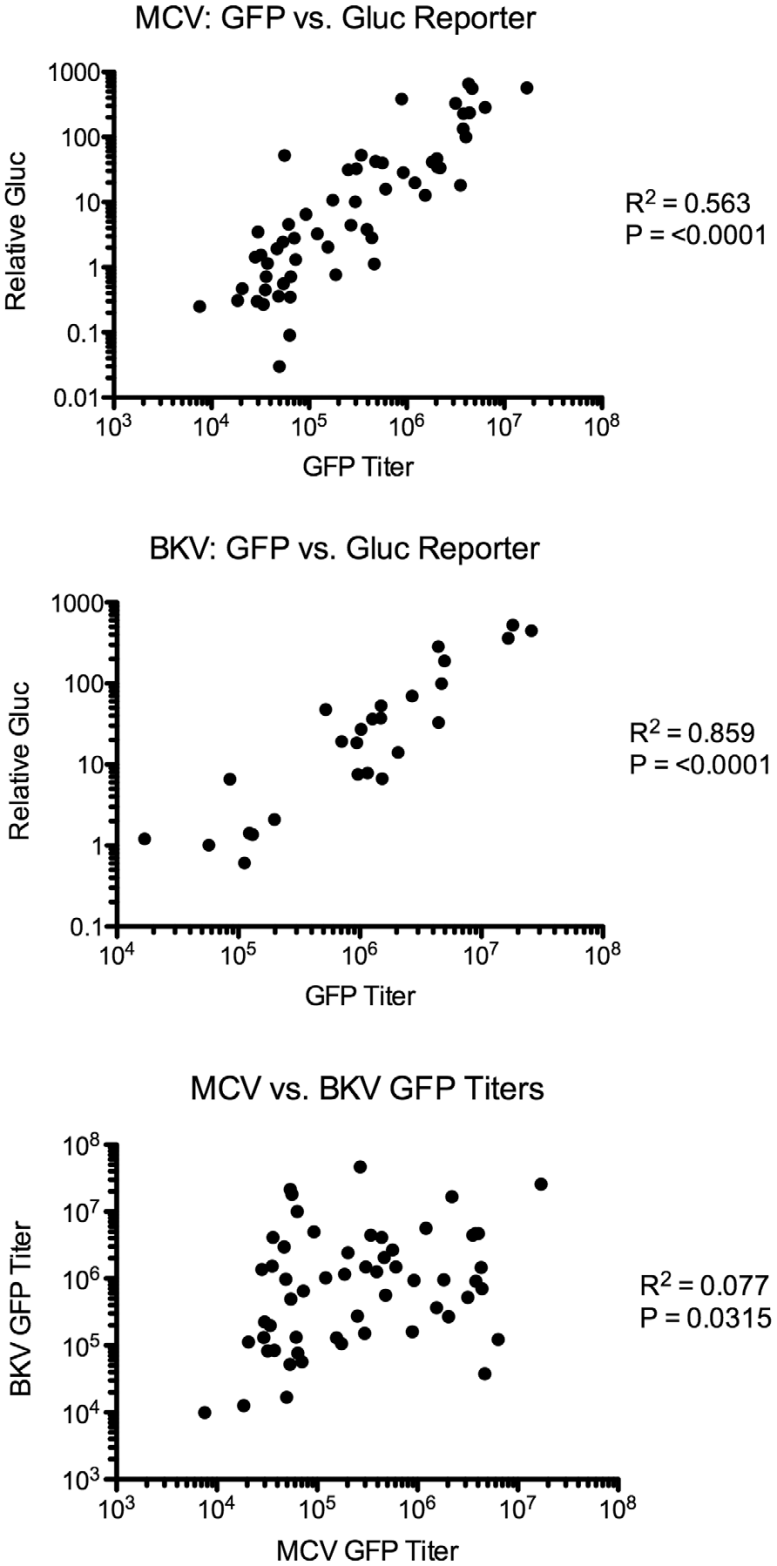
Correlations between GFP titers and relative GLuc transduction. The correlation between GFP-reporter pseudovirus titers and relative GLuc-reporter pseudovirus RLUs for each NCI-60 cell line examined with both types of pseudovirus is shown. R^2^ values were calculated with Prism software using the Pearson two-tailed correlation test. The poor correlation between MCV-GFP and BKV-GFP titers is also shown.

### Calculations of Titer and Relative Transduction

The dose of virus providing transduction levels of 5–10% (or less, if higher levels were not achievable) for each cell type was selected to calculate the viral titer of that cell type. The DTP website provides the doubling time of each of the NCI-60 cell lines. Other cells that were examined were assumed to have doubled once prior to infection. The estimated number of cells at the time of virus inoculation was multiplied by the percentage of GFP-positive cells observed at 72 hours. This value was then divided by the volume of virus stock required to achieve that rate of transduction. The resulting titer represents the number of GFP transducing units in one milliliter of virus stock. The relative transducivity of *Gaussia* luciferase reporter viruses was calculated by dividing the RLUs (background subtracted) resulting from transduction of each cell type by the RLUs from A549 cell transduction at the same dose, from the same experiment. The dose selected was a middle dose in the dilution series, which in no cell line produced the maximum detection limit of the luminometer. The quotient of each division was multiplied by 100 to give the percentage transduction relative to A549 cell transduction.

Using NCI-60 PatternMiner software, we used the GLuc and GFP data sets to search for positive correlations between cellular gene expression and relative transducibility. Consistent with the hypothesis that enhanced secretion of the GLuc reporter gene by the melanoma lines artificially inflated their apparent transducibility, top hits for the GLuc titer analysis included a large number of melanocyte-specific genes involved in secretion and melanosome biogenesis (data not shown). We therefore focused on analysis of the G

### Expression Level Quantitation of Gene Transcripts in the NCI-60 Using Five Microarray Platforms

The determination of transcript expression levels has been described previously [46], [47], [48]. In brief, probes from five platforms, the Affymetrix (Affymetrix Inc., Sunnyvale, CA) Human Genome U95 Set (HG-U95, GEO accession GSE5949); the Human Genome U133 (HG-U133, GEO accession GSE5720); the Human Genome U133 Plus 2.0 Arrays (HG-U133 Plus 2.0, GEO accession GSE32474); and the GeneChip Human Exon 1.0 ST array (GH Exon 1.0 ST, GEO accession GSE29682) and the Agilent (Agilent Technologies, Inc., Santa Clara, CA) Whole Human Genome Oligo Microarray (WHG, GEO accession GSE29288). Processing normalization was done as described previously [46].

Quality control was done based on intensity range across the NCI-60, with values less than 1.2 log_2_ dropped, and average probe/probe Pearson’s correlations, with values less than 0.30 dropped as described previously [46]. Probes that passed quality control were then transformed to z-scores, and the average z-scores determined for each gene for each cell line as described previously [46].

### Native Virus Production

MCV virions were produced as previously described [25]. Specifically, the day prior to transfection, 2.5 million 293 TT cells were plated in a 25 cm^2^ flask. The cells were co-transfected with 4 µg re-ligated MCV isolate R17a (GenBank accession number HM011555) genomic DNA as well as 4 µg each of expression plasmids carrying MCV small t antigen (pMtB) and large T antigen (pADL*) genes. Transfected cells were transferred to a 75 cm^2^ flask when crowding occurred and later transferred to two 225 cm^2^ flasks for additional space to grow. After 5–6 days, when cells in the two 225 cm^2^ flasks were nearly confluent, virions were harvested and purified using the same methods as were used for reporter viruses, except that Benzonase (Sigma) and Plasmid Safe (Epicentre) nucleases at a concentration of 0.1% replaced RNase A/T1 cocktail. BKV virions (Gardner strain, GenBank Accession number JF894228, [7]) were kindly provided by Gene Major (NINDS, NIH) [49].

**Table 2:** The top 100 genes that correlate with viral titers.

MCV transduction correlated genes						BKV transduction correlated genes					
	Gene Name	*r*		Gene Name	*r*		Gene Name	*r*		Gene Name	*r*
**1**	RUNDC3B	0.845	**51**	FMN2	0.514	**1**	DNALI1	0.776	**51**	GNMT	0.664
**2**	RPL17P4	0.814	**52**	OR14K1	0.514	**2**	OTOR	0.766	**52**	C6orf165	0.662
**3**	ZCWPW2	0.788	**53**	MSLN	0.512	**3**	CPAMD8	0.753	**53**	NCRNA00257	0.66
**4**	TCEAL2	0.745	**54**	PPP1R14A	0.51	**4**	AGXT2	0.752	**54**	PDZD2	0.658
**5**	SLC13A5	0.744	**55**	ATG9B	0.51	**5**	RSL24D1P9	0.75	**55**	ELF5	0.657
**6**	RGS7BP	0.742	**56**	GNAO1	0.507	**6**	POU2F3	0.744	**56**	C5orf58	0.656
**7**	MAGEL2	0.733	**57**	NEFHP1	0.504	**7**	PIP	0.742	**57**	AMZ1	0.655
**8**	PNMA3	0.732	**58**	C2orf49	0.5	**8**	PCP4L1	0.741	**58**	INPP5J	0.652
**9**	AHSG	0.725	**59**	CTCFL	0.497	**9**	CDC20B	0.738	**59**	ABCC6	0.651
**10**	ZNF157	0.698	**60**	CPB1	0.497	**10**	TDPX2	0.738	**60**	CCDC42B	0.641
**11**	DNAJC5G	0.687	**61**	IL1RL2	0.492	**11**	AASDHPPT	0.738	**61**	SEPP1	0.639
**12**	CEACAMP5	0.674	**62**	NEFH	0.491	**12**	PHACTR1	0.736	**62**	MFSD7	0.638
**13**	C11orf85	0.655	**63**	FAM70A	0.49	**13**	CLDN8	0.734	**63**	FKBP1AP1	0.638
**14**	SNAP91	0.633	**64**	RING1	0.483	**14**	KLF8	0.734	**64**	PIH1D2	0.637
**15**	LYVE1	0.622	**65**	CCNYL2	0.476	**15**	SERPINA6	0.734	**65**	ARHGAP40	0.631
**16**	OR10A6	0.618	**66**	PDE6B	0.47	**16**	ACER1	0.734	**66**	ADHFE1	0.628
**17**	IQSEC3	0.613	**67**	TCAM1P	0.468	**17**	TRPV6	0.732	**67**	TNKS1BP1	0.626
**18**	KBTBD12	0.612	**68**	RPS6P9	0.468	**18**	BNIPL	0.73	**68**	SPINK13	0.626
**19**	SEMA3D	0.61	**69**	GALC	0.467	**19**	PGR	0.729	**69**	C1orf88	0.624
**20**	RASIP1	0.605	**70**	SEMA3E	0.464	**20**	CYP4Z2P	0.728	**70**	C11orf52	0.624
**21**	CCR10	0.598	**71**	DCI	0.464	**21**	PDE6H	0.724	**71**	MPP7	0.622
**22**	MEI1	0.597	**72**	IGFBPL1	0.462	**22**	ABCC11	0.723	**72**	CRISP3	0.621
**23**	RFPL4A	0.594	**73**	BEX5	0.462	**23**	KRT8P16	0.723	**73**	KCTD6	0.614
**24**	ADCY5	0.59	**74**	TNNT2	0.462	**24**	ABCC12	0.723	**74**	NCAM2	0.611
**25**	C4orf44	0.588	**75**	NAP1L3	0.459	**25**	TRPV3	0.72	**75**	PLEKHG4B	0.611
**26**	KCTD8	0.584	**76**	FAM100A	0.459	**26**	XG	0.719	**76**	PCDHGA1	0.61
**27**	OR6W1P	0.579	**77**	MAEA	0.457	**27**	CYP1A2	0.714	**77**	C6orf154	0.61
**28**	ABCB1	0.578	**78**	TRBVAOR9–2	0.455	**28**	C1orf64	0.714	**78**	CALCOCO1	0.609
**29**	NLRP10	0.574	**79**	INPP4B	0.454	**29**	RTP1	0.714	**79**	RPS17P2	0.606
**30**	OR9A2	0.57	**80**	CLIP3	0.454	**30**	TRIL	0.712	**80**	C4orf19	0.6
**31**	STAG3	0.57	**81**	HCP5P14	0.45	**31**	HPX	0.712	**81**	ZMYND10	0.599
**32**	FLT4	0.558	**82**	DPH3	0.45	**32**	CYP2T2P	0.709	**82**	TTC6	0.597
**33**	RAMP2	0.556	**83**	PSG9	0.446	**33**	EGOT	0.707	**83**	RMST	0.596
**34**	ABCB4	0.556	**84**	OR5K2	0.444	**34**	C1orf168	0.706	**84**	RERG	0.595
**35**	ANKRD6	0.554	**85**	LYRM2	0.444	**35**	ABCC13	0.702	**85**	PKD1L1	0.595
**36**	ACTC1	0.553	**86**	C9orf85	0.442	**36**	PNPLA7	0.697	**86**	FLT4	0.593
**37**	HLA-L	0.552	**87**	LAMC3	0.441	**37**	C9orf150	0.695	**87**	HOXC4	0.593
**38**	GNGT2	0.541	**88**	PCLO	0.44	**38**	BACH1	0.693	**88**	SPINK5	0.593
**39**	NHLRC1	0.541	**89**	LGI3	0.438	**39**	POU6F2	0.691	**89**	SYT8	0.592
**40**	SMC1B	0.541	**90**	KRTAP4–7	0.437	**40**	PRLR	0.689	**90**	TMPRSS13	0.591
**41**	C14orf167	0.539	**91**	HDAC5	0.435	**41**	MAP6D1	0.687	**91**	TRIM17	0.587
**42**	RPS9P2	0.536	**92**	MMP27	0.432	**42**	CYP4X1	0.686	**92**	TNNI2	0.581
**43**	RPL26P13	0.535	**93**	RILPL1	0.431	**43**	CYP4Z1	0.685	**93**	PXMP4	0.578
**44**	NCRNA00107	0.534	**94**	C17orf104	0.428	**44**	BFSP2	0.685	**94**	RANBP3L	0.577
**45**	SNORA11C	0.533	**95**	AEBP1	0.427	**45**	PTGER3	0.679	**95**	HOXC6	0.577
**46**	RSPO4	0.531	**96**	SYCP1	0.425	**46**	ZNF552	0.678	**96**	SPEF2	0.576
**47**	RGS9	0.524	**97**	ZFYVE26	0.424	**47**	RPLP0P2	0.677	**97**	RLN2	0.576
**48**	TMEM174	0.523	**98**	GNAL	0.423	**48**	C10orf71	0.676	**98**	IVL	0.574
**49**	ABI3	0.521	**99**	LAMA1	0.421	**49**	DLL1	0.665	**99**	GRIK4	0.573
**50**	LDHC	0.518	**100**	C17orf72	0.421	**50**	CLIC3	0.665	**100**	C9orf24	0.573

### Native Virus Replication

The six selected MCV-transducible NCI-60 cell lines and 293–4T cells were split into a 48-well plate at a density that produced a 40–60% confluent monolayer the next day: 293–4T = 7.5×10^4^, A549/ATCC = 1.8×10^4^, SF-539 = 1.2×10^4^, SK-MEL-5 = 2.5×10^4^, MALME-3M = 5×10^4^, MDA-MB-468 = 5×10^4^, NCI/ADR-RES = 2.5×10^4^. Duplicate wells containing the sub-confluent cell monolayers were inoculated with 2.8×10^8^ MCV genome equivalents (MOI ∼8000) or with 4.0×10^7^ BKV genome equivalents (MOI ∼1000). Roughly 24 hours after virus inoculation, all wells were incubated in trypsin to resuspend the cells, and the trypsin was neutralized with growth medium. One set of cells was re-plated in a larger well for continued growth while the other set was centrifuged and resuspended in Hirt buffer I, then frozen. A total of four days after virus inoculation, the re-plated cells were collected by trypsinization and resuspended in Hirt buffer I. Plasmid DNA from all samples was isolated by modified Hirt extraction [50], and the number of genomic copies of MCV or BKV in the samples was determined by quantitative PCR as described previously [25].

## Results

A primary goal of this work is to establish positive and negative correlations between previously-established gene expression patterns in the NCI-60 cell lines and their relative transducibility with MCV and BKV pseudoviruses carrying GFP or GLuc reporter genes. Since gene expression profiles are likely to be sensitive to culture conditions, the procedures and reagents used by the DTP were mimicked as much as possible when culturing and plating cells. However, in a few instances the plating density specified by the DTP resulted in a visibly confluent monolayer at the time of virus inoculation. Infection by some DNA viruses is known to depend on cell cycle progression [51], which can be inhibited by close cellular contact. Therefore, transduction of seemingly confluent monolayers was repeated at subconfluent cell densities. In two cell lines, EKVX and HS 578T, an increased apparent titer was achieved at lower cell density. Titers at both high and low cell density are reported in Table 1 and Table S1, but for discussion and graphing of transduction efficiency, only the lower cell density titer was considered. For bioinformatics analysis of gene expression correlates (see below), the high-density titer was instead evaluated.

**Table 3:** Transducibility of other potentially relevant cell lines and primary cells.

			Viral Titer		RLUs (% of A549)	
Cell Line Name	Inoculation Density	Cell Origin	MCV-GFP	BKV-GFP	MCV-GLuc	BKV-GLuc
UISO	7500	Merkel Cell Carcinoma (MCV-)	165000	1680000	0.73	2.00
MaTi	∼10000	MCC Lymph Node Metastisis (MCV-)	0	0	0.03	0.05
WaGa	∼10000	MCC Patient Ascites (MCV+)	240000	0	0.04	0.02
MKL-1	∼10000	MCC Nodal Metastasis (MCV+)	0	0	0.07	0.03
HeLa	5000	Cervical Adenocarcinoma	160000	2160000	0.05	ND
HaCaT	7500	Transformed Keratinocyte	0	0	0.00	0.00
HEKa	3500	Primary Human Adult Keratinocyte	1113000	14952000	ND	ND
HEMn	10000	Primary Human Epidermal Melanocyte	90000	360000	0.00	0.08
PFSK-1	7500	Primitive neuroectodermal tumor	435000	577500	ND	ND

The cells listed in column one were plated in 96-well plates at the density shown the day prior to addition of MCV or BKV pseudoviruses. The viral titer of GFP-reporter pseudoviruses was determined by flow cytometry. GLuc-reporter transduction was measured by luminometry following injection of substrate. Relative light units (RLUs) are displayed as a percentage of A549 cell transduction RLUs. ND =  not determined.

All 60 of the NCI-60 cell lines were challenged with MCV and BKV pseudoviruses encapsidating mammalian expression plasmids encoding a GFP reporter gene. A dilution series of each reporter virus was added to cells that were plated approximately 20 hours prior in individual rows of a 96-well plate at the density specified in Table 1. Three days later, cells were analyzed for GFP expression by flow cytometry, and viral titers were determined based on the percentage of transduced cells. The MCV and BKV titers for each cell type are displayed in Figure 1, and cells are grouped according to their tumor origin. The data show that MCV and BKV can transduce cell lines from a broad range of solid tumor types. Strikingly, all the leukemia-derived cell lines were resistant to transduction with both MCV and BKV. Each of the seven colon cancer cell lines was resistant to MCV transduction. An ovarian cancer line named NCI/ADR-RES produced the highest MCV titer. The MCV titer on this line was more than double the titer of the next most transducible line, MALME-3M (a melanoma line). NCI/ADR-RES were also highly transducible by BKV, as were two other ovarian cancer lines, but the most BKV-transducible line was a breast cancer line called T-47D. One intriguing observation is that melanoma cell lines appeared to be over-represented in the top 25% of MCV titers, with six of the nine melanoma lines falling into the most transducible quartile. In contrast, no melanoma lines appear in the top 25% of BKV titers. As melanocytes are an abundant constituent of the skin, the result raises the possibility that MCV naturally infects melanocytes, and the apparent preference of MCV for melanoma lines might be a consequence of characteristics the lines have retained from their pre-malignant origin.

**Figure 3:**
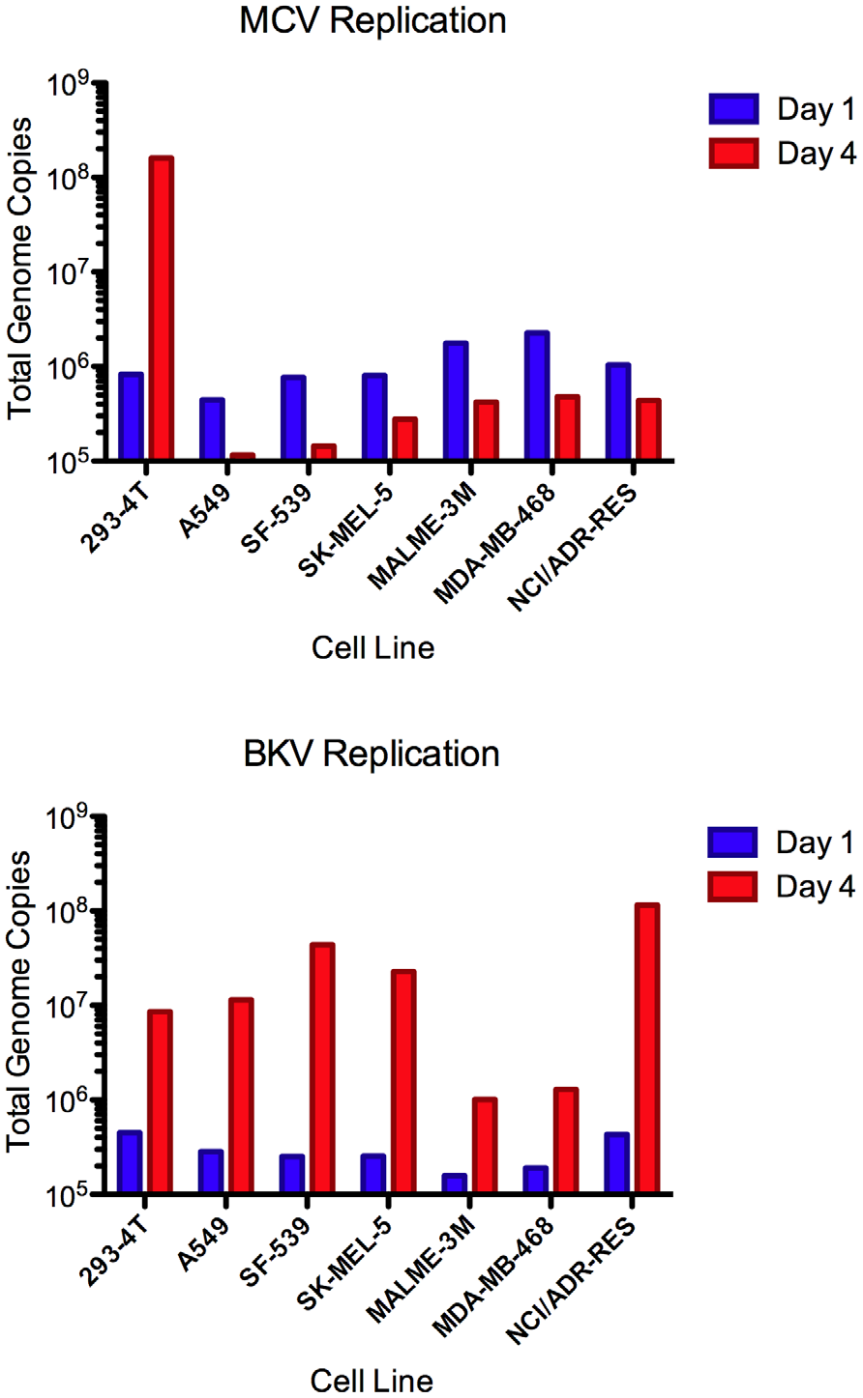
Replication of Native MCV and BKV in infected NCI-60 cells. NCI-60 cell lines with high MCV titers and 293–4 T cells were inoculated with native MCV or BKV in duplicate. The following day, one sample from each cell type was collected and frozen while the other was re-plated. On the fourth day, the second sample was collected. Low molecular weight DNA was purified and the number of MCV or BKV genomic copies was determined by quantitative PCR. One representative experiment of four is shown.

To confirm the viral titers calculated from GFP reporter vector transduction of the NCI-60 cell lines, nearly all of the cell lines were re-challenged with MCV vectors carrying an encapsidated *Gaussia* luciferase (GLuc) reporter gene. BKV carrying the GLuc reporter was also tested in a large fraction of the cell lines. The relative transduction efficiency of cell lines was calculated based on measurements of the secreted luciferase activity in the medium of cells inoculated with various dilutions of purified reporter vector three days prior. The resulting GLuc-based transduction efficiency value was highly correlated to the GFP-based viral titer determined for each cell line (Figure 2). However, the Gluc-based screen appeared to inflate the relative transducibility of melanoma cell lines, as compared to GFP-reporter titers. We believe this to be the result of increased secretory activity in these lines, as opposed to increased transduction by the Gluc reporter virus, since the GLuc reporter plasmid also appeared to drive disproportionately high GLuc expression in melanocytes when the plasmid was delivered by liposome-mediated transfection (data not shown).

FP-based data set. The top 100 positive gene correlations with MCV-GFP and BKV-GFP titers in the NCI-60 cell lines are shown in Table 2 and the full set of all correlations is listed in Table S2.

In light of reports that MCV can be isolated from the skin of individuals, and because integrated copies of the viral genome are found in a carcinoma that arises in epidermal Merkel cells, the infectability of various skin cell types is of particular interest. MCV and BKV transduction was examined in two MCV-negative MCC cell lines (UISO and MaTi) and two MCV-positive MCC cell lines (WaGa and MKL-1) [42]. Additionally, the immortalized epidermal keratinocyte line HaCaT, as well as primary epidermal keratinocytes were analyzed. Primary melanocytes were also examined. Of these cell types, only primary keratinocytes were efficiently transduced by MCV (Table 3). One MCC MCV+ and one MCC MCV- line was transduced in a dose-dependent manner, yet MCV titers on these cells were very low. HeLa, an HPV18-transformed cervical adenocarcinoma cell line, was transduced relatively poorly by MCV reporter vectors. Human primary melanocytes also showed poor transducibility with the MCV reporter vectors, casting doubt on the hypothesis that the high transducibility of melanoma lines reflects a natural tropism for non-transformed melanocytes. The other skin cell lines we examined were not transduced or they appeared to be transduced poorly, but reporter gene expression levels were not dependent on virus dose. BKV transduced most of the same skin cell types as MCV. However, BKV transduced these cells much more efficiently than MCV in each case. In fact, the BKV titer on primary keratinocytes would fall within the top 10% of NCI-60 panel titers.

A recent report examining replication and gene expression of native MCV in various cell lines found that the primitive neuroectodermal tumor cell line, PFSK-1, might represent a viable model system for production and study of the MCV virus [29]. However, viral gene expression was evaluated after transfection of MCV genomic DNA and the authors reported that it was not possible to propagate native MCV virions in the PFSK-1 line. It is conceivable that MCV propagation in PFSK-1 cells is limited by their infectability. We therefore examined the transduction efficiency of PFSK-1 cells using our GFP-reporter viruses and determined titers for MCV and BKV pseudoviruses. Both viruses transduced PFSK-1 cells at a moderate level. The MCV titer in PFSK-1 cells was just above the median titer for all NCI-60 cell lines, while the BKV titer was just below the median (Table 3). Thus, it does not appear that the failure of MCV to establish a spreading infection in PFSK-1 cultures is strictly due to a block at the level of infectious entry.

Previous reports have shown that expression of viral genes from the native MCV genome is highly restricted in all cell lines so far tested [25], [28], [29]. This innate block can be partially overcome using a cell line, named 293–4 T, which stably expresses the small t and large T antigen proteins of MCV *in trans*. The 293–4 T system can be used for production of native MCV virions and analysis of MCV infectivity [25]. In order to determine if NCI-60 cells that are readily transduced by MCV reporter vectors might also support native MCV gene expression and replication, we selected a set of highly MCV-transducible cell lines from differing tissue types, and treated each line with native MCV virions. MCV replication was determined by comparing a baseline qPCR measurement of MCV genome copy number at day 1 post-infection to the MCV copy number at day 4 post-infection. Although replication of the MCV genome resulted in a robust increase in copy number between days 1 and 4 in 293–4 T cells, the other relatively transducible lines showed a decline in MCV copy number, suggesting a failure of the virus to replicate robustly in these lines (Figure 3). In contrast, the culture-adapted BKV Gardner isolate showed detectable replication between days 1 and 4 in each of the lines.

## Discussion

Cells capable of growing in culture are indispensable to the study of viruses. Generally, researchers seek to identify cell lines that represent “relevant” models for the cell types a virus is thought to productively infect in vivo. Although this approach can be successful, there are also examples of viruses infecting cultured cells from tissues the virus is not known to infect in vivo. Conversely, the natural target cell type can become resistant to infection during the process of adaptation to in vitro culture. For example, papillomaviruses, which have a narrow tropism for keratinocytes in vivo, are unable to infect primary keratinocytes cultured in vitro [52].

For MCV, the search for a relevant cell type is a particular challenge since the cellular tropism of MCV in vivo is not yet known. Given the fact that MCV was discovered in a form of skin cancer and is abundantly shed from the surface of apparently healthy skin surfaces, it is tempting to speculate that MCV, like papillomaviruses, productively infects keratinocytes. However, it is also possible to imagine that less-abundant skin cell types, such as melanocytes, white blood cells, or Merkel cells, become productively infected with MCV.

In this report, we show that the initial steps of MCV infection (penetration of the cell membrane and delivery of encapsidated DNA to the nucleus) are readily completed in primary keratinocytes, but very inefficient in primary melanocytes. Puzzlingly, the reverse is true for the immortalized keratinocyte line HaCaT, which is impervious to MCV entry, while a majority of melanoma lines are highly permissive. MCV pseudoviruses readily delivered reporter genes to tumor cell lines derived from tissues where MCV has not been abundantly detected, such as the brain or prostate [53], [54]. Taken together, our results show that MCV infectious entry is governed by cellular factors that are altered during the process of immortalization, transformation, and/or adaptation to culture. Analyses of cultured cell lines therefore cannot provide a reliable answer to the question of which cell types MCV naturally infects in vivo.

For some viruses, such as HIV, tissue culture tropism provides a strong indication of cellular tropism in vivo (reviewed in [55]). This is likely due to the restricted nature of the expression of the HIV entry receptors. For other viruses, such as HTLV-1, in vivo tropism appears to be far more restricted than in vitro tropism (reviewed in [56]). Three different receptors mediate HTLV-1 infection, and while two of the molecules are believed to be ubiquitous, the third is often up-regulated in tumor cells [57]. Thus, the fact that MCV can enter a wide range of different tumor types in vitro does not necessarily imply that the virus can infect a similarly wide range of different tissue types in vivo. Continuously growing monolayer cultures of tumor cells have often proven to be poor models of in vivo tumor behavior (reviewed in [58]), and cells undergo numerous changes on the path to tumor development. Specifically, studies have shown that cell transformation can bring about changes in the synthesis and expression glycosaminoglycans (GAGs) [59]. As MCV attachment to cells is mediated by GAG binding, changes in GAG expression might also have influenced the outcome of our NCI-60 analysis. Binding to each of the lines was not investigated, and differences in GAG expression would likely affect the transduction efficiency by MCV regardless of co-receptor expression or other regulatory factors. Indeed, there is great potential for multiple regulating factors that are differentially expressed in each cell type to confound the bioinformatics analysis and identification of vital entry factors. Furthermore, approximately 1/3 of the gene transcript levels in the NCI-60 cell lines do not significantly correlate with protein levels [37], which could result in many false positives and false negatives in our analysis.

Despite the possible theoretical barriers to bioinformatic identification of cellular factors essential for MCV infectious entry, we made a limited pilot attempt to identify essential factors using an siRNA knockdown approach. Genes were selected from preliminary bioinformatic analyses using the publicly accessible COMPARE tool at the DTP website (http://dtp.nci.nih.gov/compare/). Selected genes were high-ranking in our analyses, a plausible role for the gene product in MCV entry could be imagined based on available information, and the correlation of gene expression with MCV entry efficiency was verified graphically. Targeted genes included *PLXNA1, CSPG4, DNAJC13, HMCN1, GDAP1, GPNMB, CAPN3*, and *SLC37A3*. In the cases of *PLXNA1* and *CAPN3* as well as *BACE2* we also attempted to disrupt protein function with semaphorins, calpeptin or β-secretase inhibitors III and IV, respectively. Although we did not reproducibly observe specific effects on MCV transduction for any of these candidate genes, our initial pilot efforts in this area should not be viewed as comprehensive. MCV seems to enter cells very slowly and asynchronously (data not shown). The roughly 72 hour time course needed to effectively measure peak transduction significantly complicates many entry experiments. For example, extended pharmacologic intervention often results in toxicity by this time and the effects of siRNA knockdown effects may wane. Nonetheless, we hope that by publishing these data that other groups may find them of value in the search for important cellular molecules mediating MCV infection.

## Supporting Information

Table S1 **Relative transducibility of NCI-60 Cell Lines.**
(XLS)

Table S2 **Correlations of viral titers with the complete set of genes.**
(XLS)
